# Transmembrane signal transduction by cofactor transport†

**DOI:** 10.1039/d1sc03910e

**Published:** 2021-08-20

**Authors:** Istvan Kocsis, Yudi Ding, Nicholas H. Williams, Christopher A. Hunter

**Affiliations:** Department of Chemistry, University of Cambridge Lensfield Road Cambridge CB2 1EW UK herchelsmith.orgchem@ch.cam.ac.uk; Department of Chemistry, University of Sheffield Sheffield S3 7HF UK

## Abstract

Information processing and cell signalling in biological systems relies on passing chemical signals across lipid bilayer membranes, but examples of synthetic systems that can achieve this process are rare. A synthetic transducer has been developed that triggers catalytic hydrolysis of an ester substrate inside lipid vesicles in response to addition of metal ions to the external vesicle solution. The output signal generated in the internal compartment of the vesicles is produced by binding of a metal ion cofactor to a head group on the transducer to form a catalytically competent complex. The mechanism of signal transduction is based on transport of the metal ion cofactor across the bilayer by the transducer, and the system can be reversibly switched between on and off states by adding cadmium(ii) and ethylene diamine tetracarboxylic acid input signals respectively. The transducer is also equipped with a hydrazide moiety, which allows modulation of activity through covalent conjugation with aldehydes. Conjugation with a sugar derivative abolished activity, because the resulting hydrazone is too polar to cross the bilayer, whereas conjugation with a pyridine derivative increased activity. Coupling transport with catalysis provides a straightforward mechanism for generating complex systems using simple components.

## Abstract 2

Synthetic transducers transport externally added metal ion cofactors across the lipid bilayer membrane of vesicles to trigger catalysis of ester hydrolysis in the inner compartment. Signal transduction activity is modulated by hydrazone formation.

## Graphical abstract

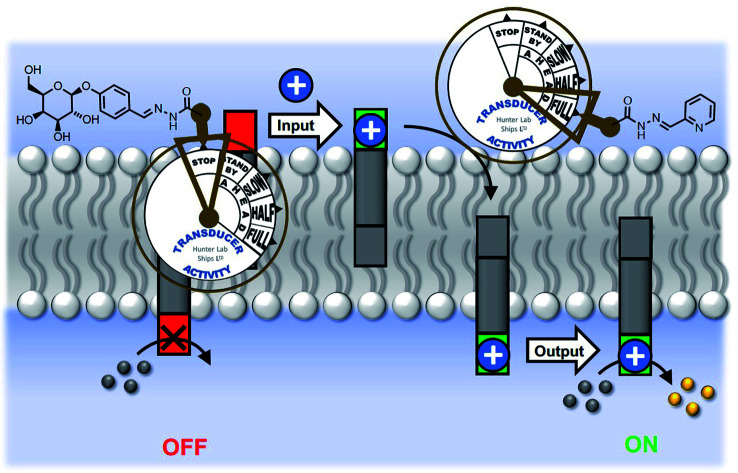

## Introduction

Cascade reaction pathways are a characteristic of biological systems through which complex functions are accomplished. Cellular communication, for instance, takes place *via* interconnected signalling and amplification cascades.^1^ In order to achieve this, biological cells synergistically couple several processes both simultaneously and subsequently using mainly protein mediators.^2^ However, designing artificial systems that can mimic the complexity of such processes is not trivial. Synthetic liposomes have proven to be useful models of some properties of natural cells, allowing the study of both biological and synthetic components at the lipid bilayer interface.^3^ The hydrophobic environment of a bilayer acts as a localization point for hydrophobic molecules,^4^ providing a quasi-two-dimensional matrix and raising the apparent concentration of membrane-bound molecules in comparison to the total bulk solution.^5,6^ This property has been exploited in catalysis^7^ and molecular imprinting.^8–10^ The compartmentalization afforded by liposomes has been used to study signal transduction,^11–13^ cascade reactions^14–16^ and transport phenomena.^17^ More recently, the concept of synthetic molecular motors and switches has been used in conjunction with liposomes to induce passive^18^ or active^19^ actions within the system. Multifunctional membrane molecules have been used to design complex synthetic systems capable of self-reproduction^20^ or recognition-based transmembrane signalling.^21^

We have recently reported a new class of membrane molecules that act as signal transducers across lipid bilayers.^22–24^ The membrane-anchored molecules are equipped with two different switchable head groups which allow them to transmit chemical signals across the lipid bilayer membranes of vesicles by a membrane translocation mechanism. Coupling of an external stimulus to a change in the polarity of one of the head groups was used to control the location of the transducer molecules in the lipid bilayer. Complexation of metal ions on the inside of the vesicles by the second head group lead to formation of a catalyst, which hydrolysed an ester substrate to generate the amplified output signal.

**Scheme 1:**
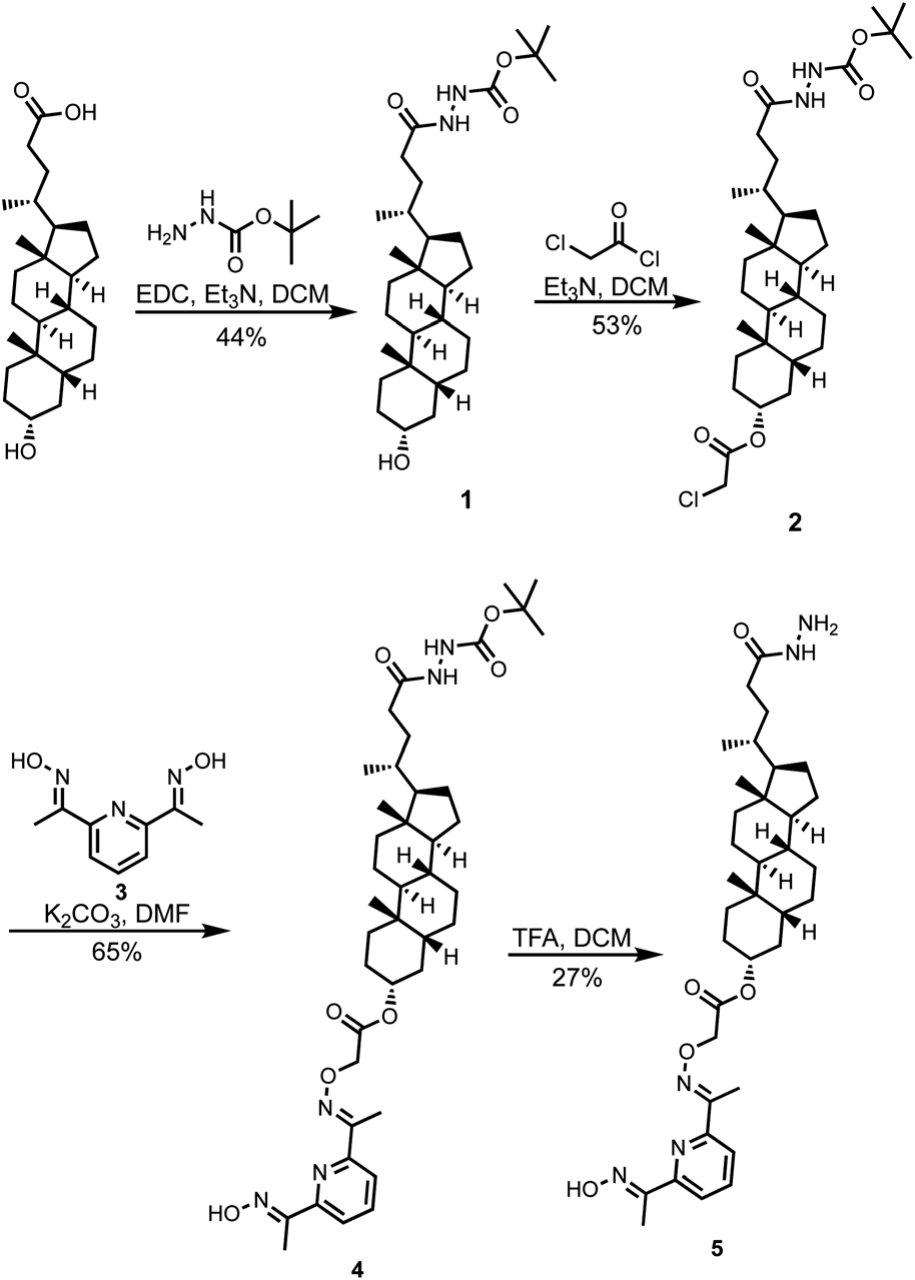
Synthesis of **5**.

Here, we describe a different signal transduction mechanism, which is based on transport of an externally added metal ion cofactor across a lipid bilayer membrane to generate a catalyst for substrate hydrolysis on the inside of vesicles. Scheme 1 shows the structure of the transducer molecule **5**. The steroid core acts as a membrane anchor to embed the compound in the lipid bilayer of vesicles, and the pyridine-oxime head group is a switchable catalyst, which hydrolyses esters when bound to a transition metal ion. The acyl hydrazide head group was designed as a point for covalent attachment of different aldehydes by hydrazone formation in order to regulate the properties of the catalytic head group.^25–28^ Hydrazones can be formed both in organic and aqueous solutions and are stable at neutral pH.^29–32^ The presence of the hydrazide therefore provides a handle for straightforward modification of the properties of the system and for *in situ* functionalisation in vesicles.^33^ However, we have found that compound **5** is able to transduce chemical signals across a lipid bilayer without any modification.

## Results and discussion

### Synthesis

**Scheme 2:**
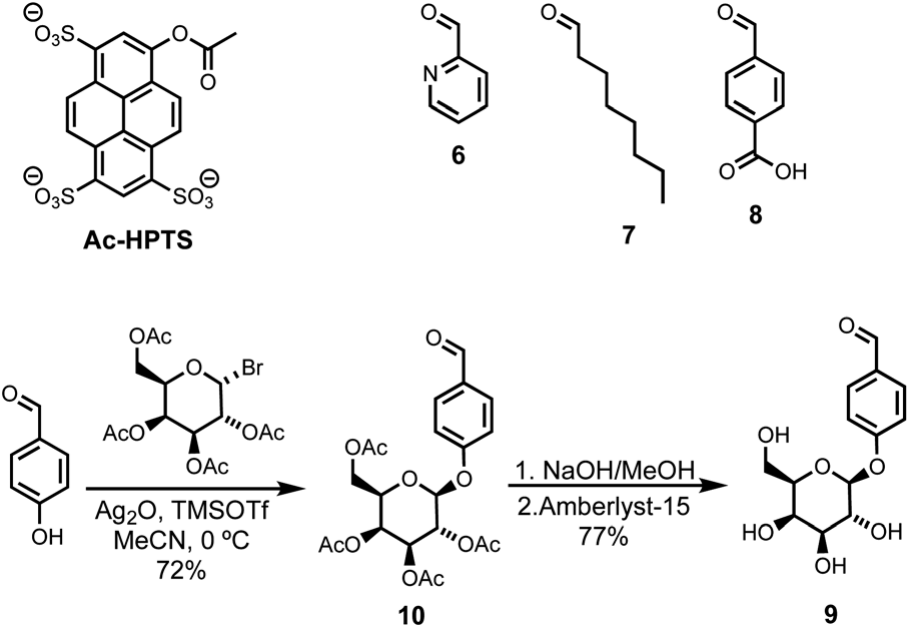
Chemical structures of the ester substrate Ac-HPTS and aldehyde derivatives **6–9**.

Compound **5** was synthesized starting from lithocholic acid as shown in Scheme 1. An 1-ethyl-3-(3-dimethylaminopropyl)carbodiimide (EDC) mediated coupling with *t*-butyl carbazate, followed by condensation with chloroacetyl chloride gave **2**. Substitution with pyridine-oxime derivative **3** and subsequent deprotection of the hydrazide gave **5**. Pyridine-oxime **3** and the ester substrate (Ac-HPTS in Scheme 2) were synthesized as previously described.^22^ The aldehyde derivatives **6**, **7** and **8**, which were used to conjugate the hydrazide moiety are commercially available, and the sugar derivative **9** was prepared using the route shown in Scheme 2.

### Transmembrane signalling

Initial assessment of catalytic properties of **5** was carried out in aqueous solution with no vesicles present. When **5** was added to a buffered solution containing Ac-HPTS, no change in fluorescence was observed over time (see Fig. S6†). Repeating this experiment in the presence of Cd^2+^ resulted in a steady increase in fluorescence intensity, which indicates hydrolysis of the ester to give the fluorescent product HPTS. This result shows that complexation of Cd^2+^ by the pyridine-oxime head group of **5** leads to a catalytically active complex. A pH of 6.6 ensures that the pyridine-oxime head group is protonated and inactive, but the metal complex is deprotonated for nucleophilic catalysis. We have previously used to Zn^2+^ pyridine-oxime complex to catalyse this reaction, but we found a significantly higher activity for the Cd^2+^ complex (see Fig. S6†).

**Fig. 1:**
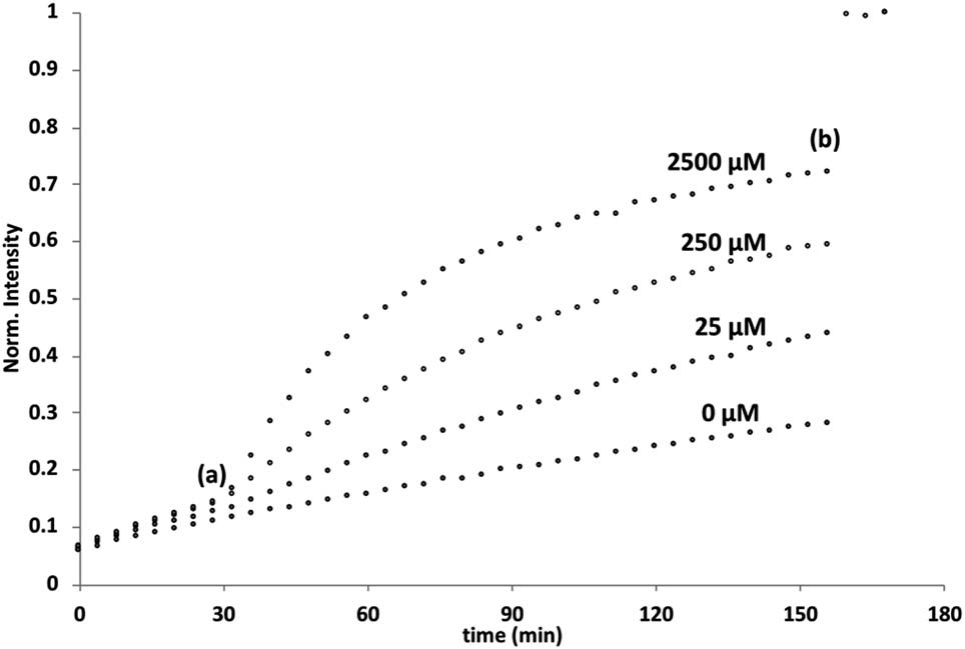
External addition of Cd^2+^ to DOPC vesicles containing 2.5 mol% **5** embedded in the lipid membrane and 250 μM Ac-HPTS encapsulated in the inner compartment at pH 6.6 with 50 mM MES buffer and 100 mM NaCl. At (a) CdSO_4_ (0–2500 μM) was added, and at (b) Triton-X 100 and NaOH were added to attain complete substrate hydrolysis. The fluorescence emission intensity due to the hydrolysis product HPTS is shown (*λ*_ex_ = 415 nm, *λ*_em_ = 510 nm).

The behaviour of **5** embedded in the bilayer of DOPC vesicles was then investigated. Vesicles were prepared containing **5** in the lipid membrane and Ac-HPTS encapsulated in the inner compartment. When external Cd^2+^ was added to the vesicles, a significant increase in fluorescence was observed, which indicates hydrolysis of the substrate is taking place on the inside of the vesicles. Fig. 1 shows that addition of increasing amounts of external Cd^2+^ to the vesicle suspension increases the rate of internal substrate hydrolysis. These results indicate that addition of Cd^2+^ to the outside of the vesicles results in formation of a catalytically active pyridine-oxime complex, which is able to hydrolyse the substrate on the inside of the vesicles. In other words, **5** must transport Cd^2+^ across the bilayer.

**Fig. 2:**
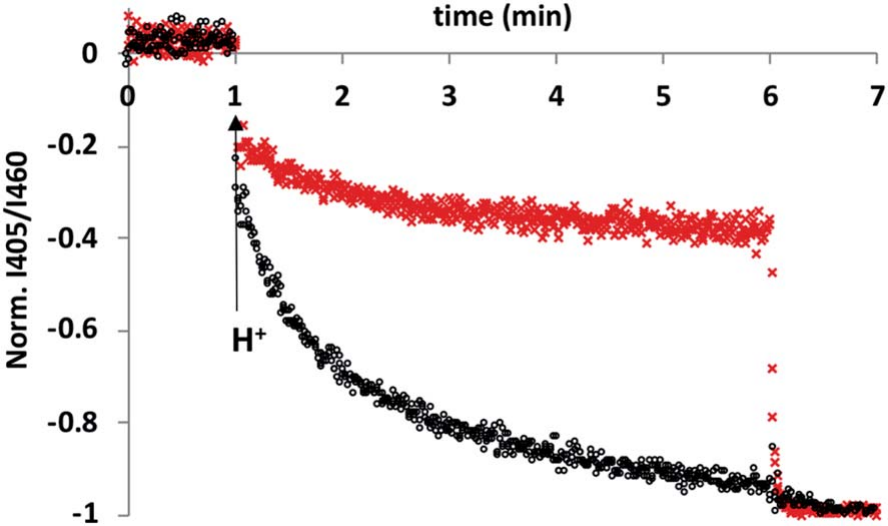
Comparison of the effect **5** (black) and of DMSO (red) on cadmium transport across vesicle bilayer membranes. DOPC vesicles containing 1 mM HPTS were prepared at pH 6.6 and suspended in 10 mM MES buffer with 100 mM CdSO_4_ and either **5** or DMSO. At *t* = 1 min, H_2_SO_4_ was added to lower the external pH to 6.1, and the local pH inside the vesicles was monitored using the ratio of the fluorescence emission at 510 nm due to the phenol (*λ*_ex_ = 405 nm) and phenolate (*λ*_ex_ = 460 nm) forms of HPTS. At *t* = 6 min, the vesicles were lysed by addition of Triton-X 100 to obtain the HPTS emission at a pH of 6.1.

To test this hypothesis, an HPTS transport assay was used.^30^ Vesicles were prepared at pH 6.6 with CdSO_4_ on both the inside and the outside of the vesicles and with HPTS encapsulated on the inside. Addition of H_2_SO_4_ to the external solution was used to generate a pH gradient across the bilayer, and changes in the internal pH of the vesicles was monitored using the fluorescence emission of HPTS (the ratio of the emission intensities at 510 nm and 460 nm provides a direct measure of local pH). Fig. 2 shows the results obtained in the presence and absence of **5** in the bilayer membrane. In the control experiment (addition of just DMSO), there was a small jump in the fluorescence emission on addition of external H_2_SO_4_, but subsequent changes were small. In the presence of **5**, the change in internal pH was much larger, and no additional change in pH was observed on lysing the vesicles with detergent, which shows that the internal pH had equilibrated to match the external pH. These results are characteristic of ion transport across the bilayer. The possible transport pathways that would allow equilibration of pH across the lipid bilayer are Cd^2+^/H^+^ antiport, Cd^2+^/OH^−^ symport, OH^−^/SO_4_^2−^ antiport and H^+^/SO_4_^2−^ symport. However, the high dehydration energy associated with sulfate transport (Δ*G* = +1080 kJ mol^−1^)^31^ means that the SO_4_^2−^ mediated pathways are very unlikely. This experiment therefore suggests that **5** transports Cd^2+^, which is consistent with the results of the catalysis experiment shown in Fig. 1.

**Fig. 3:**
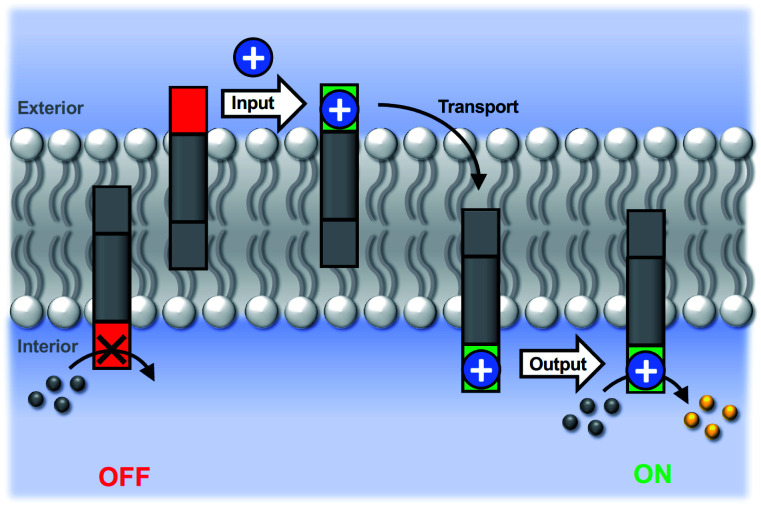
Transmembrane signal transduction by cofactor transport. In the resting OFF state, the pyridine-oxime head group (red) is inactive. The input signal is a metal ion cofactor (blue circle) added to the external vesicle solution. Binding to the pyridine-oxime head group of the membrane-embedded transducer in the outer leaflet leads to transport of the metal ion across the bilayer to the inner compartment. The metal complex of the pyridine-oxime head group (green) formed in the inner leaflet catalyses hydrolysis of an ester substrate (grey balls) encapsulated on the inside of the vesicles to generate a fluorescent product (yellow balls) as the output signal of the ON state.

Fig. 3 illustrates the signal transduction mechanism that accounts for the results in Fig. 1 and 2. Transducer **5** sits in the lipid bilayer, due to the steroid core, which provides a hydrophobic membrane anchor. The pyridine-oxime head group can access both the outer and inner aqueous solutions. When cadmium ions are present in the external solution, binding to the pyridine-oxime head group leads to transport of the metal ion across the bilayer. The active complex could be the singly charged 1 : 1 complex, the neutral 2 : 1 complex, or transport could proceed *via* a relay process where one transducer in the outer leaflet passes a metal ion to another transducer in the inner leaflet, but these mechanisms are difficult to distinguish. Regardless of the precise mechanism, the result is that externally added cadmium ions appear in the inner compartment of the vesicles. Formation of the catalytically active Cd^2+^ complex of the pyridine-oxime head group on inner surface of the vesicle bilayer leads to hydrolysis of the substrate inside the vesicles.

### Hydrazone modulation of activity

**Fig. 4:**
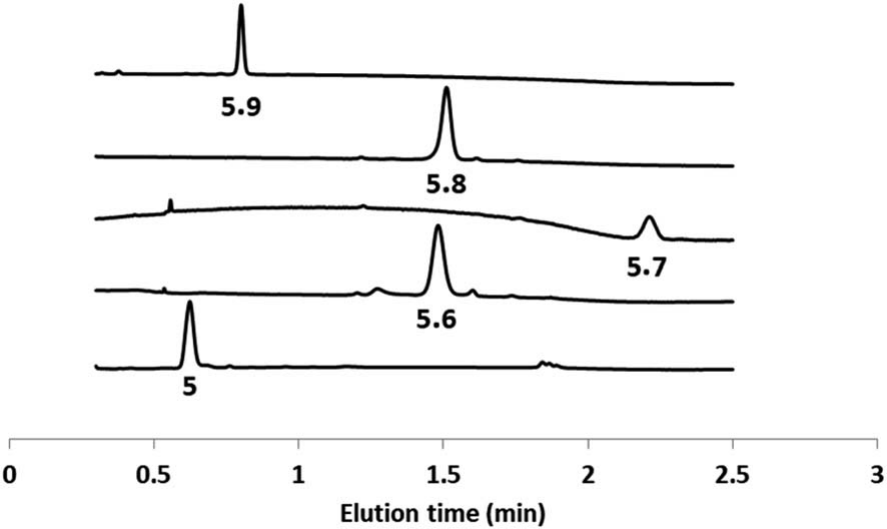
HPLC-UV traces (absorption at 280 nm) of DOPC vesicles containing **5** or the hydrazone conjugates formed with aldehydes **6–9**.

The second head group on **5** is an acyl hydrazide, which provides a site for conjugation of aldehydes and an opportunity for modulation of catalytic activity by formation of hydrazone head groups of different polarity. Starting from compound **5**, hydrazones were formed either prior to incorporation into vesicles or by *in situ* functionalisation of vesicles containing **5**. The reactions were quantitative, and Fig. 4 shows HPLC traces of vesicle suspensions containing the hydrazone conjugates of **5** obtained using the four different aldehydes shown in Scheme 2. The hydrazones are stable in the aqueous conditions used for these experiments, and no traces of the starting hydrazide **5** were observed in any case. The *in situ* conjugation reactions were much faster with aldehydes **6**, **7** and **8** (complete conversion in less than half an hour) compared with **9**, presumably due to the polarity of the sugar group, which limits access of **9** to the membrane anchored hydrazide.

**Fig. 5:**
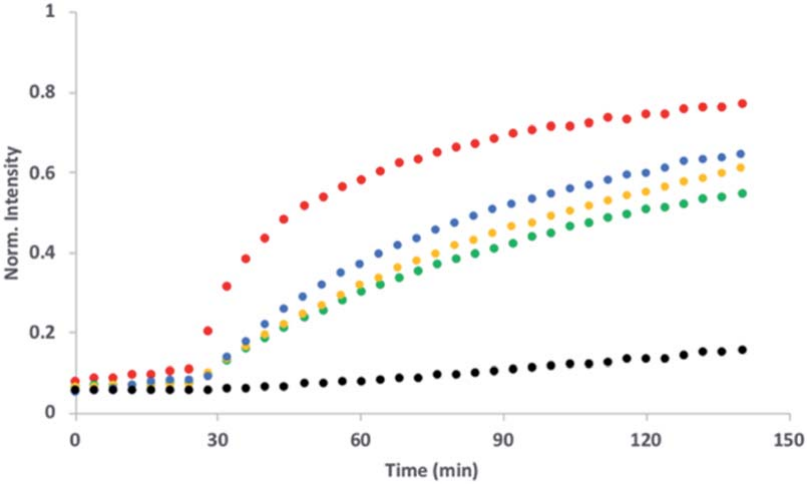
Effect of aldehyde conjugation on transmembrane signal transduction. At *t* = 30 min, 1 mM CdSO_4_ was added to DOPC vesicles containing 250 μM Ac-HPTS encapsulated in the inner compartment at pH 6.6 with 50 mM MES buffer and 100 mM NaCl and with 2.5 mol% of different transducers embedded in the lipid bilayer: the parent hydrazide **5** (blue) or the hydrazone conjugates formed with aldehydes **6** (red), **7** (yellow), **8** (green) or **9** (black). The fluorescence emission due to the hydrolysis product HPTS is shown (*λ*_ex_ = 415 nm, *λ*_em_ = 510 nm).

Fig. 5 shows signal transduction experiments carried out with the hydrazone conjugates. Vesicles were prepared containing the hydrazone embedded in the lipid membrane and Ac-HPTS encapsulated in the inner compartment. After 30 minutes, Cd^2+^ was added to the external solution of the vesicle suspension. As observed previously for **5**, in the absence of Cd^2+^, there was no significant change in fluorescence with time, but addition of Cd^2+^ lead to catalysis of substrate hydrolysis. Fig. 5 shows that the nature of the aldehyde moiety has a dramatic effect on activity. Conjugation of **5** with the sugar aldehyde **9** completely abolished signal transduction. The benzoic acid and *n*-heptyl hydrazones show similar activity to the parent hydrazide, and the pyridyl hydrazone shows enhanced activity.

Additional control experiments were carried out to investigate the possible role of the pyridyl hydrazone in increasing the activity of the system. Compound **4** has a protected hydrazide and cannot form hydrazones. When compound **4** was used in place of **5**, the rate of the catalysed reaction was unaffected by the addition of aldehyde **6** (see Fig. S7†). Thus the enhanced activity observed with **6** is not due to metal ion transport or catalysis by the aldehyde itself. When the Cd^2+^ transport experiment shown in Fig. 2 was repeated using the hydrazone conjugate formed by **5** and **6**, the rate of transport was the same as that observed for **5** alone (see Fig. S8†). These experiments suggest that the pyridyl hydrazone increases the rate of the catalytic reaction and does not increase the rate of transport by chelating metal ions.

**Fig. 6:**
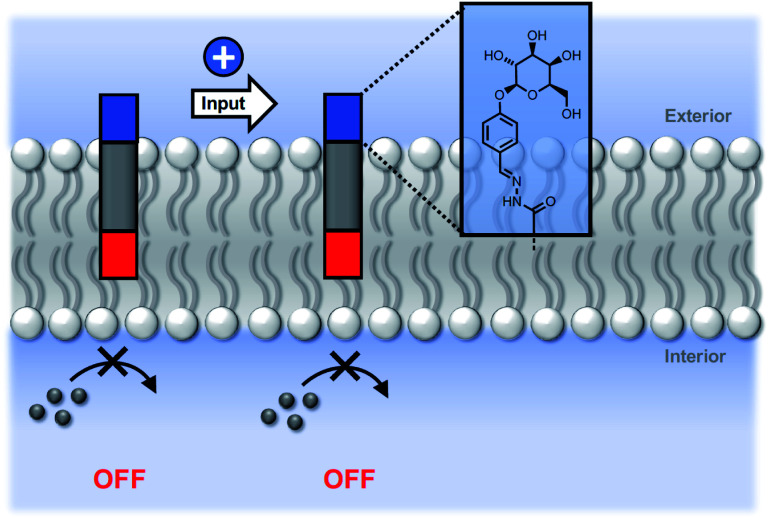
Modulation of catalytic activity by hydrazone formation. The sugar head group locates preferentially in the aqueous phase, forcing the pyridine-oxime head group inside the lipid bilayer, and blocking any interaction with the metal ion cofactor.

We conclude that the modulation of activity observed in Fig. 5 is due to the effect of the hydrazone head groups on the accessibility of the pyridine-oxime complex to the inner vesicle solution (Fig. 6). The hydrophilic sugar moiety in **9** holds the hydrazide head group in the aqueous solution, preventing the transducer from crossing the bilayer and forcing the catalytic head group into the membrane. As result, the metal ion complex is not formed in the inner compartment, and there is no substrate hydrolysis. The benzoic acid and *n*-heptyl head groups appear to have little effect of the distribution of the transducer in the membrane or on the accessibility of the catalytic head group to the inner compartment. The enhanced activity associated with the pyridyl head group is presumably due to a change in the way in which the transducer sits at the membrane interface that increases the activity of the catalyst in the inner leaflet.

### Reversible ON/OFF switching

**Fig. 7:**
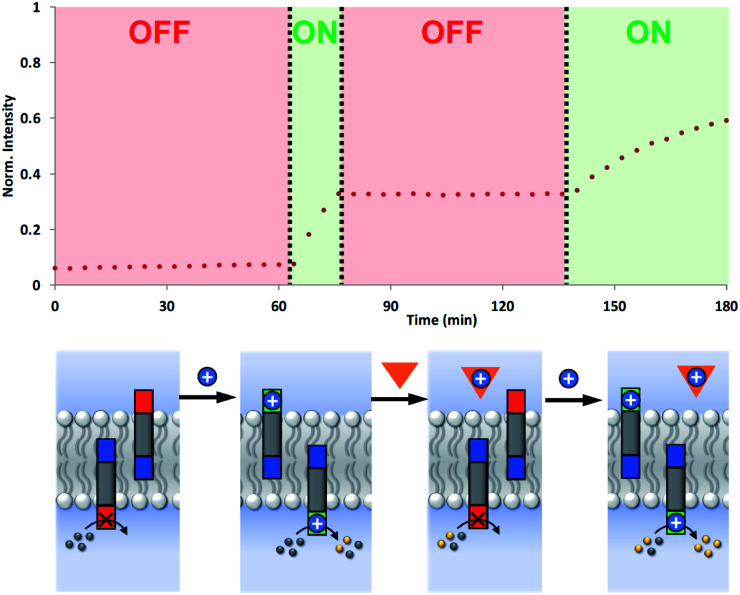
Reversible ON–OFF switching of signal transduction using the hydrazone conjugate formed by **5** and **6** (2.5 mol% in DOPC vesicles containing 250 μM Ac-HPTS encapsulated in the inner compartment at pH 6.6 with 50 mM MES buffer and 100 mM NaCl). In the resting state, there is no hydrolysis of the ester substrate inside the vesicles. Addition of Cd^2+^ (blue circle) to the external vesicle solution initiates catalysis in the inner compartment of the vesicles, giving an ON state. Subsequent addition of EDTA (orange triangle) to the external vesicle solution removes the Cd^2+^ from the transducer head group turning the system OFF. Addition of more Cd^2+^ to the external vesicle solution turns the system back ON again. The fluorescence emission intensity due to the hydrolysis product HPTS is shown (*λ*_ex_ = 415 nm, *λ*_em_ = 510 nm).

The coupling of cofactor transport with catalysis represents a novel mechanism for transmembrane signal transduction. Catalytic generation of the hydrolysis product on the inside of the vesicles is switched on by the presence of metal ions added to the outside of the vesicles. It should therefore be possible to make this system reversible by raising and lowering the external metal ion concentration. Fig. 7 shows experiments on the most active system, the hydrazone derivative formed by conjugation of **5** and **6**. Catalysis on the inside of the vesicles is switched on by adding Cd^2+^ to the external vesicle solution and then switched off again by external addition of EDTA. The transducer transports Cd^2+^ into the internal compartment of the vesicles, where the activated catalytic head group hydrolyses the ester substrate. When EDTA is added externally, the transducer transports the Cd^2+^ back out of the vesicles and delivers it to the EDTA, which cannot cross the bilayer. The removal of Cd^2+^ deactivates the catalytic head group and halts hydrolysis of the substrate. Subsequent addition of more Cd^2+^ to the external vesicle solution switches the catalysis on the inside of the vesicles back on again. The rate of increase in fluorescence emission intensity appears slower in the second ON phase, but this reflects the consumption of substrate.

## Conclusions

The intricate functional diversity of biological systems comes with the drawback of structural complexity and stability issues once removed from their natural environment. While proteins are extremely efficient in their tasks, use of proteins in non-biological systems is challenging. Synthetic analogues capable of achieving similar functions are desired, as they can be prepared more easily and tailored to specific uses. Here we report the synthesis of a membrane molecule capable of conjugation with aldehydes while embedded in vesicle membranes, transport of transition metal cations and concomitant catalysis of ester hydrolysis. We demonstrate that the catalytic activity can be modulated, depending on the aldehyde used for conjugation. Significant amplification of hydrolytic activity was registered when hydrazone **6** was generated, while in the case of hydrazone **9** no activity was observed. We have further shown that the hydrolytic activity of vesicle systems containing **5** and Ac-HPTS can be controlled by turning the system on and off by loading/unloading of liposomes with Cd^2+^.

The multifunctional nature of the compounds presented here open new avenues for the design of complex biochemical tools. The catalytic function can be exploited to trigger drug release from vesicles^34^ while at the same time making use of the hydrazone formation for implementation in post injection repurposing of drugs.^35^ Similarly, the dual transport/catalysis property can be exploited for the design of nanoreactors,^16,36^ where compartmentalization *via* liposomes can lead to easily controllable sequence reactions as well as simple purification methods for the products.

## Data availability

All supporting data is provided in the ESI.†

## Author contributions

IK, CAH and NHW devised the experiments, YD synthesised compound **9**, IK carried out the experiments, and all authors contributed to the manuscript.

## Conflicts of interest

There are no conflicts to declare.

## Supplementary Material

SC-012-D1SC03910E-s001
