# Supplementary material for: Transmembrane signal transduction by cofactor transport
Source: Chem Sci. 2021 Aug 20;12(37):12377–82. doi: 10.1039/d1sc03910e (PMC8480319; doi:10.1039/d1sc03910e)
Supplement: SC-012-D1SC03910E-s001 [file SC-012-D1SC03910E-s001.pdf]

Electronic Supplementary Material (ESI) for Chemical Science.
This journal is © The Royal Society of Chemistry 2021

Transmembrane signal transduction by cofactor transport

Istvan Kocsis,[a] Yudi Ding,[a] N. H. Williams[b] and Christopher A. Hunter [a,*]

[a] *Yusuf Hamied Department of Chemistry, University of Cambridge, Lensfield Road, Cambridge CB2 1EW, U.K.*

[b] *Department of Chemistry, University of Sheffield, Sheffield S3 7HF, U.K.*

Supplementary Information

Table of Contents

1. General information S2

2. Synthesis S3

3. Hydrazone characterisation S9

4. Ester hydrolysis experiments S14

5. Cadmium transport experiments S16

1. General information

^1H and ^{13}C NMR NMR spectra were recorded on a 400-MHz Bruker® spectrometer. Chemical shifts are reported as δ values in ppm. Flash chromatography was carried out on an automated system (Combiflash® Rf+ LumenTM) using pre-packed cartridges of silica (25 µm PuriFlash® Column) or neutral alumina (50 µm RediSep® Rf Column). GPC purification of the vesicles was carried out using GE Healthcare PD-10 desalting columns prepacked with Sephadex® G-25 medium. Fluorescence spectra were recorded using a Cary Eclipse fluorescence spectrophotometer (Agilent Technologies) in Hellma® Analytics Suprasil® quartz cuvettes. pH measurements were conducted using a Mettler-Toledo SevenCompactTM pH meter equipped with an InLab® Micro electrode. Vesicles were assembled in Eppendorf® polypropylene ProteinLoBind® polypropylene microcentrifuge tube and extruded as described below using Avanti® Polar Lipids extruder kits, equipped with Avestin® LiposoFast Liposome Factory 200 nm polycarbonate membranes with GE Healthcare Whatman® 10 mm polyester filter support. Solutions or vesicles suspensions were transferred using Eppendorf Multipette® Xstream Pippette with Combitips Advanced® or Hamilton Microliter™syringes. All reagents and solvents were used without further purification. Chemicals were purchased from Sigma-Aldrich® and used without further purification.

2. Synthesis and characterization

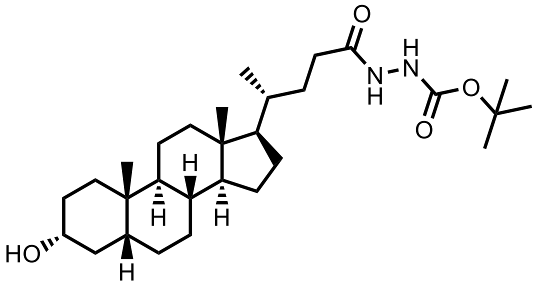

1

Lithocholic acid (1.129 g, 3 mmol), triethylamine (1.39 mL, 10 mmol) and *N*-(3-dimethylaminopropyl)-*N'*-ethylcarbodiimide hydrochloride (630 mg, 3.3 mmol) were suspended in dichloromethane (50 ml). *tert*-Butyl carbazate (0.792 mg, 6 mmol) was added and the mixture was left to stir overnight at room temperature. The organic phase was washed with brine (3x20 ml) and dried over $MgSO_4$. Purification by silica gel chromatography (combiflash, 0-5% MeOH in dichlorometane) afforded a white solid (645 mg, 44%).

^{1}H-NMR (400 MHz, ($CDCl_3$) δ (ppm): 7.24 (br, 1H), 6.52 (br, 1H), 3.65 (m, 1H), 2.30 (m, 1H), 2.13 (s, 1H), 1.99-0.94 (m,40H), 0.66 (s, 3H). ^{13}C -NMR (100 MHz, $CDCl_3$) 172.99, 155.54, 81.80, 71.87, 56.48, 55.94, 42.76, 42.10, 40.43, 40.18, 36.46, 35.85, 35.43, 35.35, 34.58, 31.27, 31.00, 30.55, 28.15, 27.20, 26.42, 24.21, 23.38, 20.83, 18.36, 12.06

HR-MS (ESI+): Calculated for $[M + Na]^+$ $[C_{29}H_{50}O_4N_2Na]^+$ m/z = 513.3663; found m/z = 513.3673.

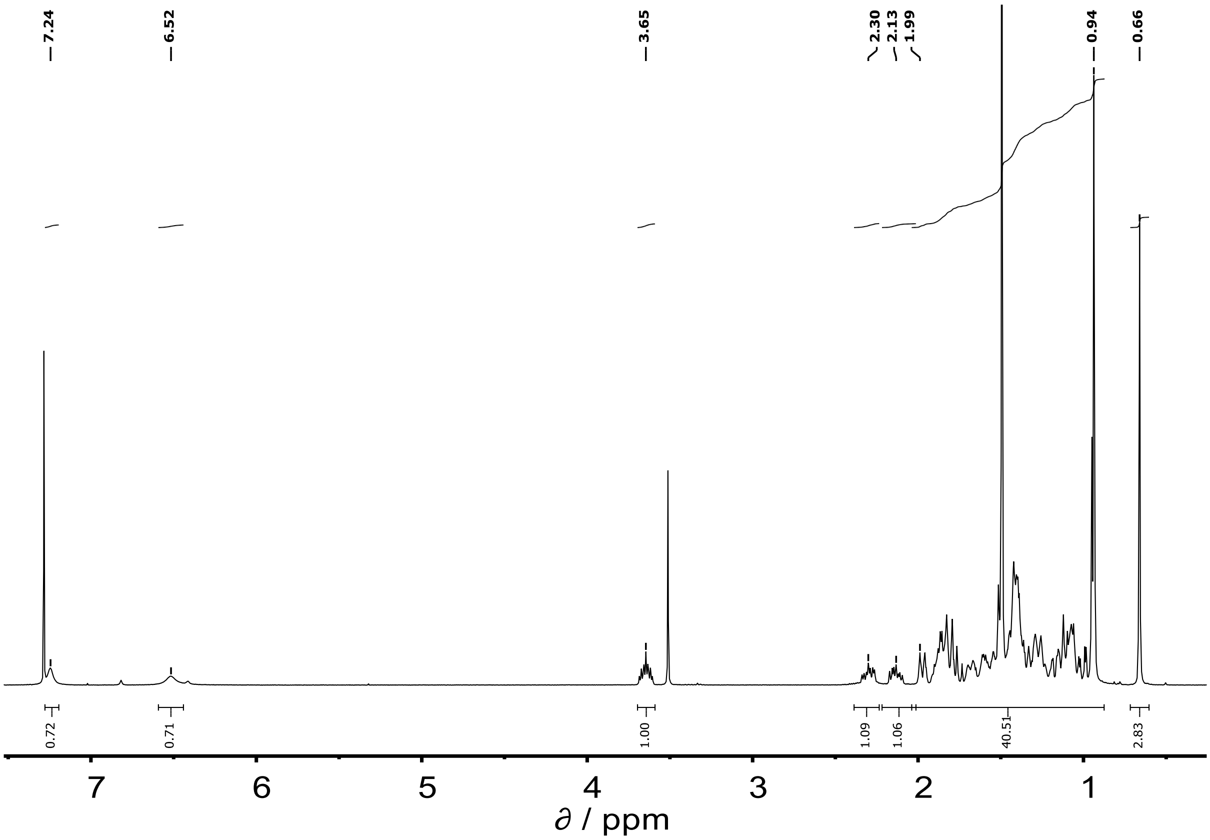

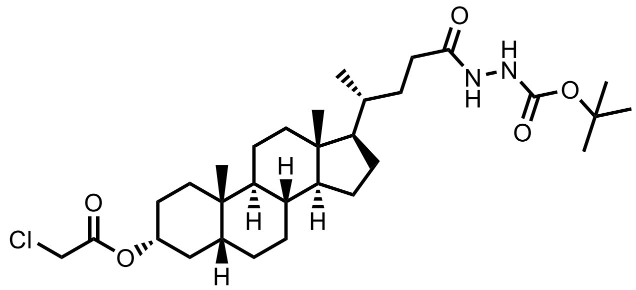

2

1 (230 mg, 0.47 mmol) and triethylamine (71 µl, 0.51 mmol) were suspended in dry dichloromethane (20 ml) and cooled down to 0°C. Chloroacetylchloride (40 µl, 0.51 mmol) was dissolved in dry dichloromethane (1 ml) and added dropwise. The solution was left to stir overnight at room temperature. The organic phase was washed with brine (3x20 ml) and dried over $MgSO_4$. Purification by silica gel chromatography (combiflash, 0-5% MeOH in DCM) afforded the product as an off white solid (149 mg, 65%).

^{1}H-NMR (400 MHz, DMSO) δ (ppm): 9.44 (br, 1H), 8.63 (br, 1H), 4.71 (m, 1H), 4.34 (s, 2H), 2.09-0.88 (m, 42H), 0.62 (s, 3H). ^{13}C -NMR (100 MHz, DMSO) δ (ppm): 172.57, 167.20, 154.99, 79.36, 76.06, 56.27, 55.37, 42.72, 41.79, 41.57, 35.76, 35.29, 34.83, 34.61, 32.12, 30.52, 28.52, 27.00, 26.55, 26.39, 24.29, 23.42, 20.87, 18.74, 12.33.

HR-MS (ESI+): Calculated for $[M + Na]^+$ $[C_{31}H_{51}O_5N_2{}^{35}ClNa]^+$ m/z = 589.3379; found m/z = 589.3386.

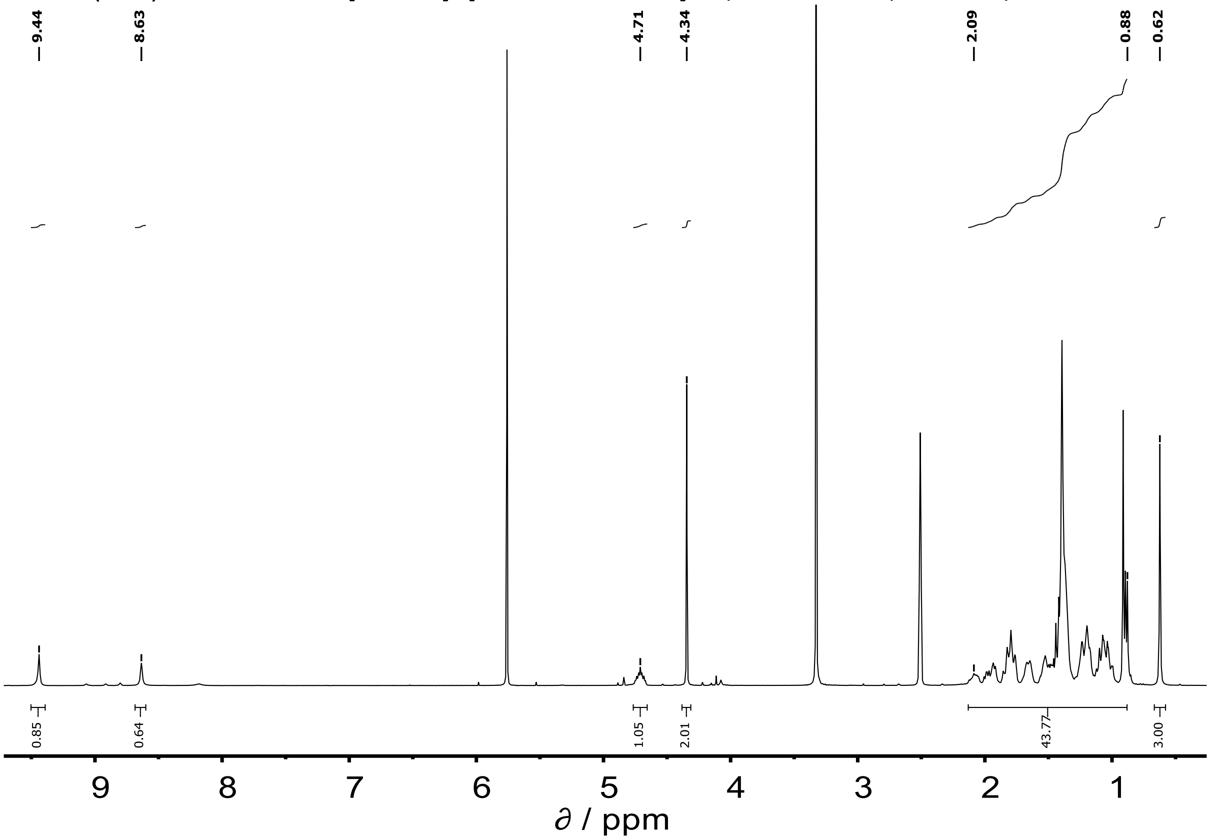

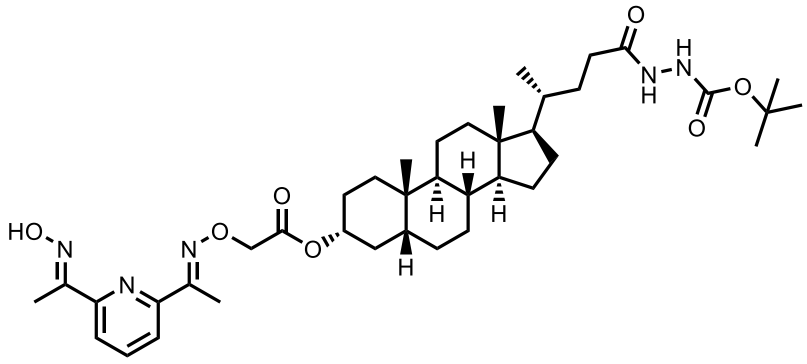

4

To a suspension of K_2CO_3 (91 mg, 0.66 mmol) in dimethylformamide (3 ml) was added **3** (25.5 mg, 0.132 mmol) and left to stir at room temperature for 10 minutes. **2** (25 mg, 0.044 mmol) was added and the solution was left to stir overnight at room temperature. The solution was diluted with $CHCl_3$ (20 ml) and washed with 5% LiCl solution (5x20 ml). The organic phase was dried over $MgSO_4$ and the solvent removed in vacuo. Purification by silica gel chromatography (combiflash, 30-60% EtOAc in petroleum ether) afforded the product as a white solid. (13.2 mg, 53%).

^{1}H-NMR (400 MHz, $CDCl_3$) δ (ppm): 9.37 (br, 1H), 7.90-7.84 (m, 2H), 7.64 (t, 1H), 6.78 (br, 1H), 4.83-4.75 (m, 3H), 2.45 (s, 3H), 2.42 (s, 3H), 2.33-2.09 (m, 2H), 1.87-0.87 (m, 40H), 0.59 (s, 3H). ^{13}C -NMR (100 MHz, $CDCl_3$) δ (ppm): 173.72, 169.81, 157.65, 156.84, 153.34, 152.75, 136.27, 120.11, 119.93, 82.26, 75.10, 71.23, 56.49, 55.30, 42.55, 41.57, 40.39, 40.12, 35.55, 34.99, 34.79, 34.39, 31.92, 31.04, 28.15, 26.82, 26.31, 26.15, 24.09, 23.23, 20.71, 18.25, 11.96, 11.23, 10.39.

HR-MS (ESI+): Calculated for [M + H]$^+$ [$C_{40}H_{62}O_7N_5$]$^+$ m/z = 724.4644; found m/z = 724.4669.

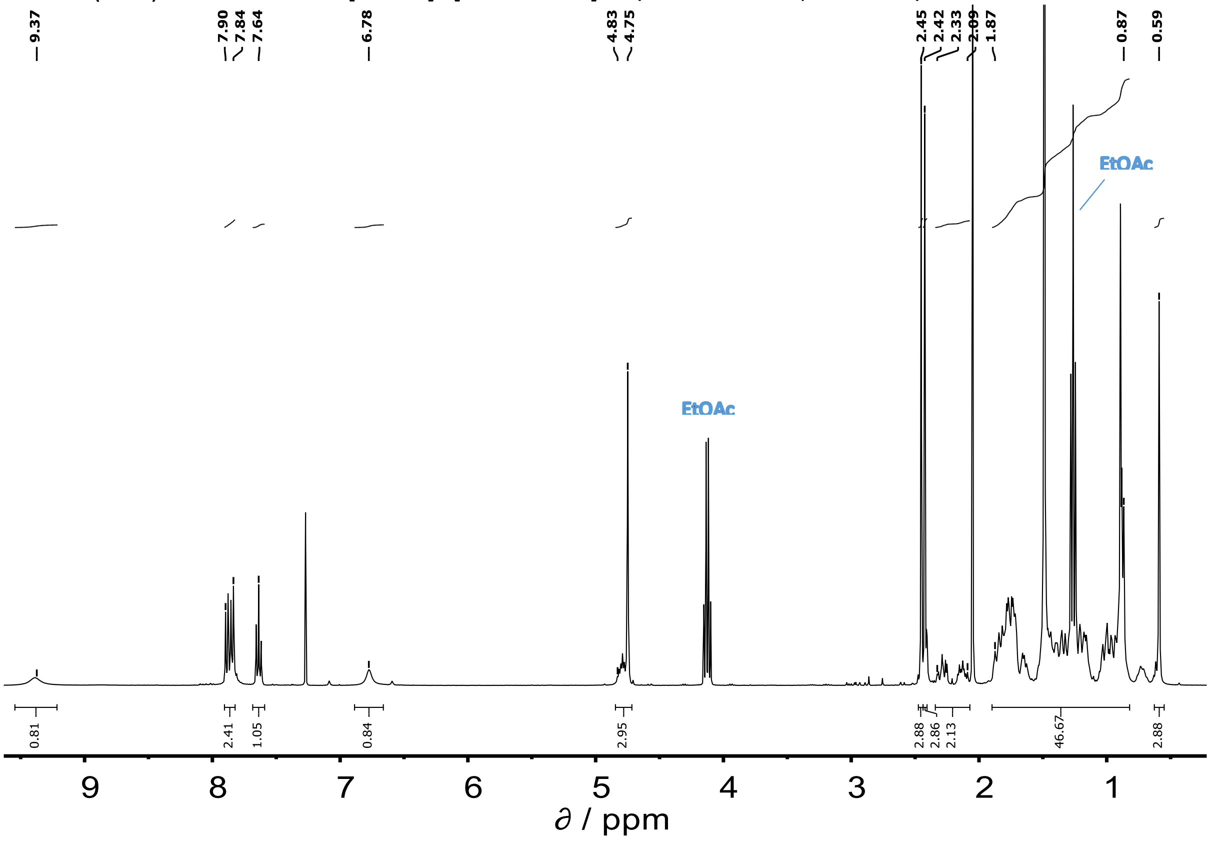

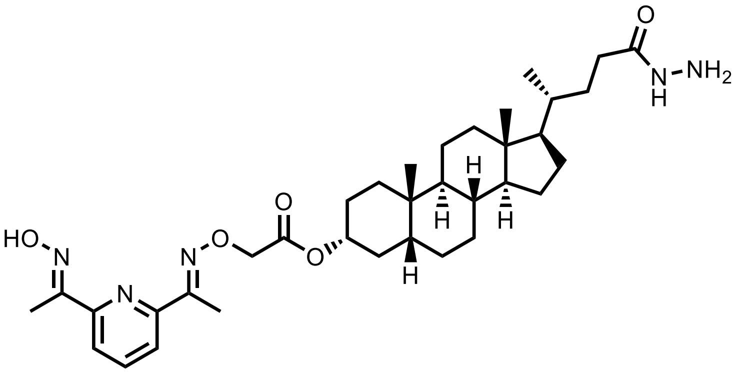

5

4 (13.2 mg, 0.018 mmol) was dissolved in dry dichloromethane (1 ml) and cooled to 0°C. Trifluoroacetic acid (1 ml) was added and the reaction was left to warm up to room temperature and left to stir for 30 min. The solvents were removed under *vacuo* and the obtained slurry was dissolved in DCM (10 ml) and washed with a saturated solution of $NaHCO_3$ (3x10 ml). The organic phase was dried over $MgSO_4$ and the solvent removed in *vacuo* to afford the afforded the product as a white solid. (10 mg, 77%).

^{1}H-NMR (400 MHz, $CDCl_3$) δ (ppm): 7.88-7.82 (m, 2H), 7.63 (t, 1H), 7.22 (br, 1H), 4.85-4.74 (m, 3H), 2.45 (s, 3H), 2.41 (s, 3H), 2.28-2.04 (m, 2H), 1.91-0.61 (m, 31H), 0.59 (s, 3H). ^{13}C -NMR (100 MHz, $CDCl_3$) δ (ppm): 174.93, 169.81, 157.46, 156.77, 153.54, 152.88, 136.25, 120.20, 120.00, 75.19, 71.23, 56.65, 55.34, 42.59, 41.62, 40.52, 40.19, 35.56, 35.12, 34.81, 34.40, 31.95, 31.20, 30.59, 28.22, 26.81, 26.37, 26.16, 24.09, 23.25, 20.79, 18.21, 11.99, 11.26, 10.34.

HR-MS (ESI+): Calculated for $[M +Na]^+$ $[C_{35}H_{53}N_5O_5Na]^+$ m/z = 646.3943; found m/z = 646.3943.

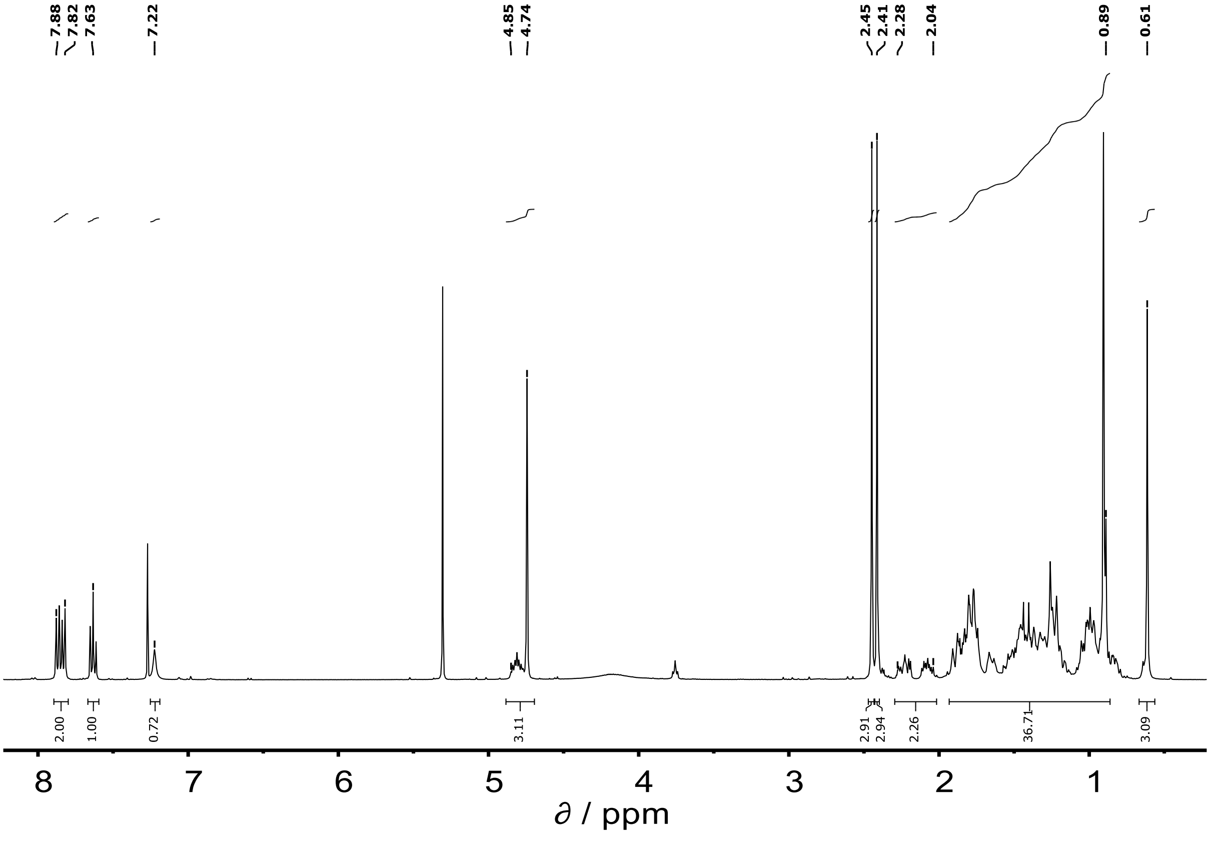

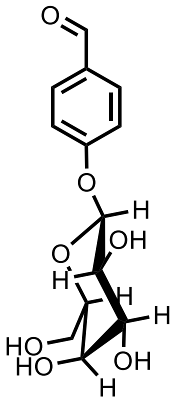

9

A solution of compound **10** (70 mg, 0.22 mmol) and sodium methoxide (12 mg, 0.22 mmol) in methanol (1 mL) was stirred at room temperature for 1.5 h. The reaction mixture was diluted with methanol (5 mL) and neutralized with Amberlyst-15 resin. The solution was poured into diethyl ether (50 mL), and the precipitate was filtered and dried to give the product as a white amorphous solid (34 mg, 77%).

^{1}H NMR (400 MHz, D_2O) δ (ppm): 9.83 (s, 1H), 7.97 (d, J = 8.6 Hz, 2H), δ 7.97 (d, J = 8.8 Hz, 1H), 7.29 (d, J = 8.8 Hz, 1H), 5.23 (d, J = 7.7 Hz, 1H), 4.04 (d, J = 3.3 Hz, 1H), 3.95 (t, J = 6.2 Hz, 1H), 3.89 – 3.86 (m, 1H), 3.82 (m, 1H). ^{13}C NMR (100 MHz, D2O) δ (ppm): 195.0, 162.0, 132.6, 130.7, 116.5, 99.8, 75.6, 72.4, 70.3, 68.4, 60.6.

HR-MS (MS+): calcd. for $C_{13}H_{16}O_7$: 284.0896, found: 284.0897.

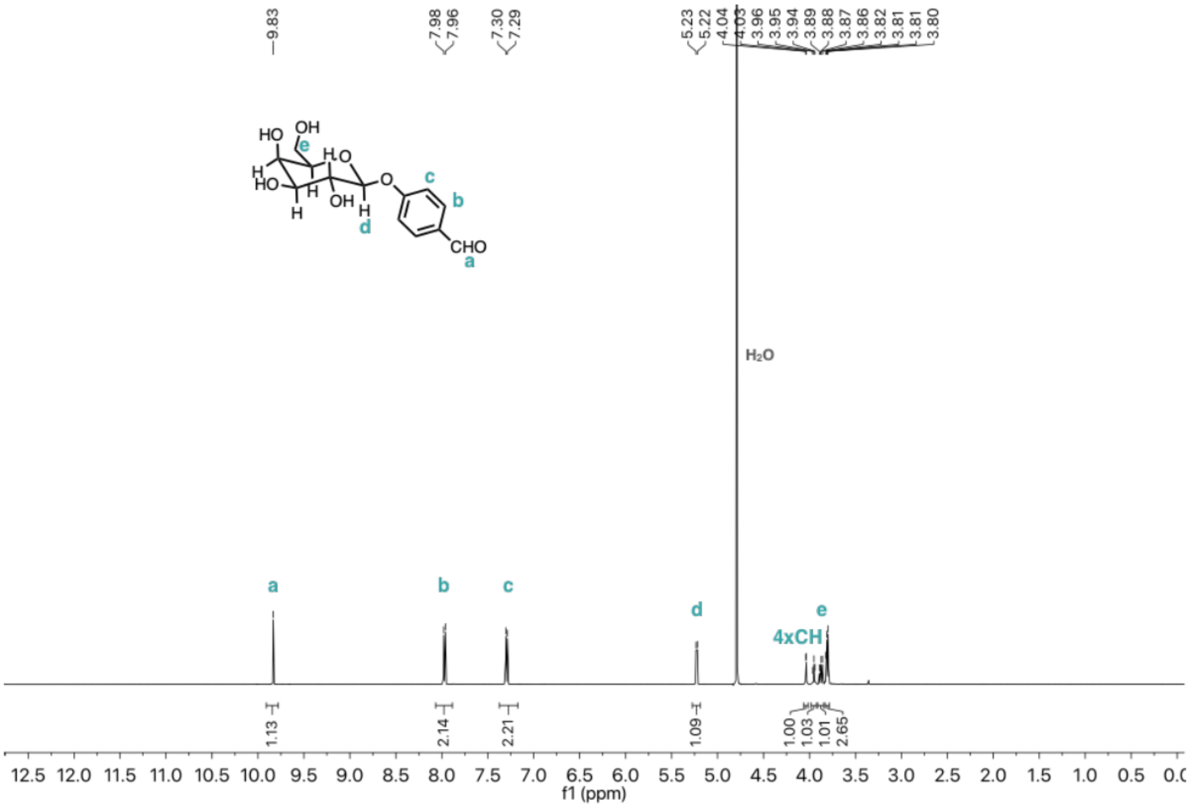

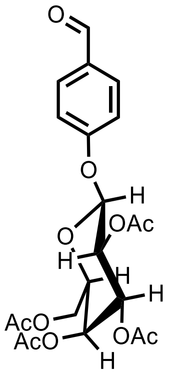

10

To a solution of acetobromo-α-D-galactose (400 mg, 0.972 mmol), 4-hydroxybenzaldehyde (118 mg, 0.972 mmol) in anhydrous chloroform (2 mL) was added silver oxide (224 mg, 1.94 mmol) with external cooling and the reaction mixture was stirred at room temperature overnight. The reaction mixture was diluted with ethyl acetate (20 mL), washed with hydrochloric acid (1 M), sodium bicarbonate (sat. aq.), brine, and dried over magnesium sulfate. The crude was purified by flash chromatography (silica, 0 – 40% ethyl acetate in petroleum ether) to yield the product as a colourless oil (396 mg, 72%).

^{1}H NMR (400 MHz, $CDCl_3$) δ (ppm): 9.93 (s, 1H), 7.87 – 7.84 (m, 2H), 7.12 -7.10 (m, 2H), 5.55 – 5.47 (m, 2H), 5.18 – 5.12 (m, 2H), 4.26 – 4.10 (m, 3H), 2.19 (s, 3H), 2.07 (s, 3H), 2.02 (s,3H). ^{13}C NMR (100 MHz, $CDCl_3$) δ (ppm): 190.6, 170.3, 170.1, 170.0, 169.3, 161.3, 131.8, 131.8, 116.7, 98.6, 71.3, 70.6, 68.4, 66.7, 61.3, 20.7, 20.6, 20.6, 20.6.

HR-MS (MS+): calcd. for $C_{21}H_{24}O_{11}$: 452.1319, found: 452.1313.

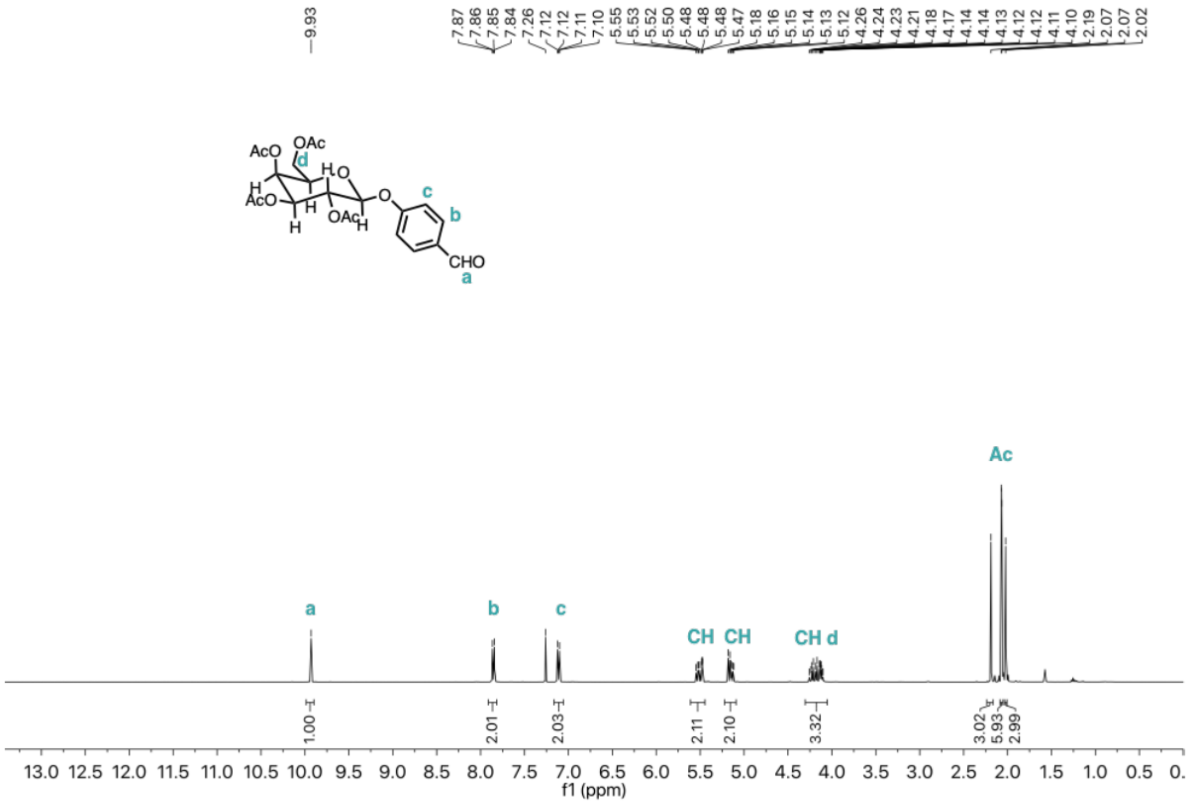

3. Hydrazone Characterisation

Crude reaction mixtures obtained by mixing **5** with different aldehydes were monitored by LCMS Waters Acquity H-class UPLC coupled with a single quadrupole Waters SQD2 with the conditions as follows: UPLC Column ACQUITY UPLC HSS T3, 100Å, 1.8 µm, 2.1 mm X 50 mm; solvent A: Water + 0.1% formic acid; solvent B: acetonitrile + 0.1% formic acid; column temperature of 40 °C.

LCMS Method:
Gradient: 0 - 2 min 5% – 100%B
2 - 3 min 100% B
Flow rate: 0.6 ml/min

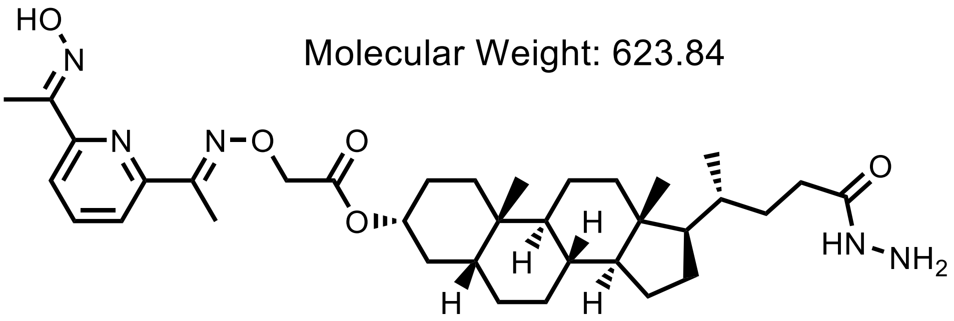

5

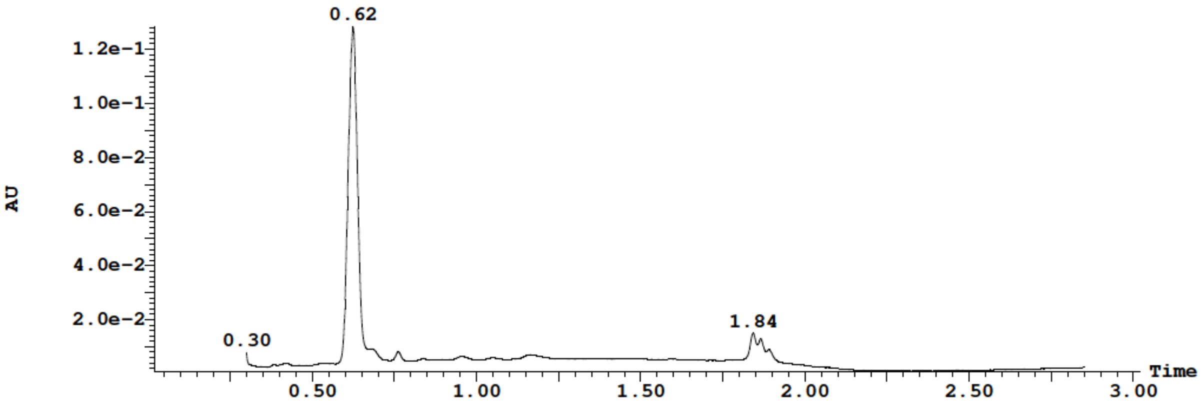

Peak ID	Compound	Time	Mass Found
5		0.64	Not Found

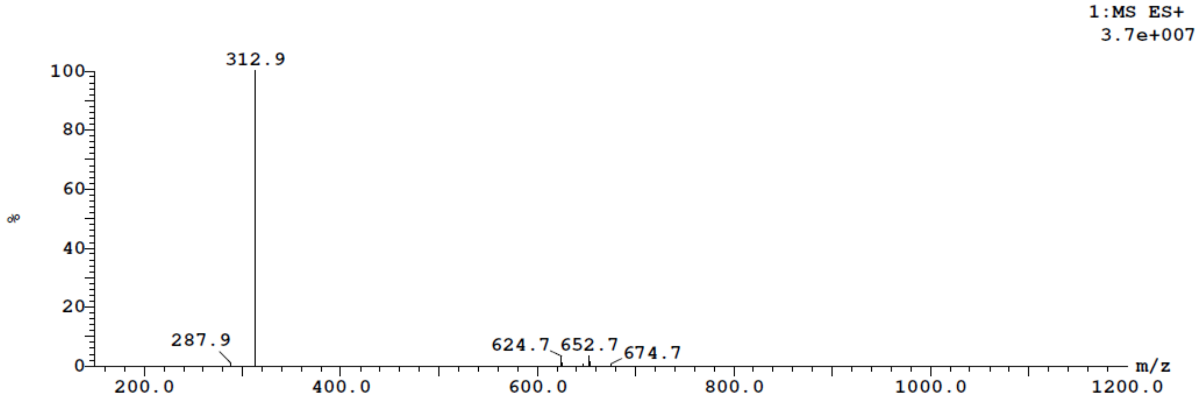

Figure S1. (top) HPLC-UV trace (280 nm) of membrane molecule **5.** (bottom) MS chromatogram of peak identified at t=0.64 min.

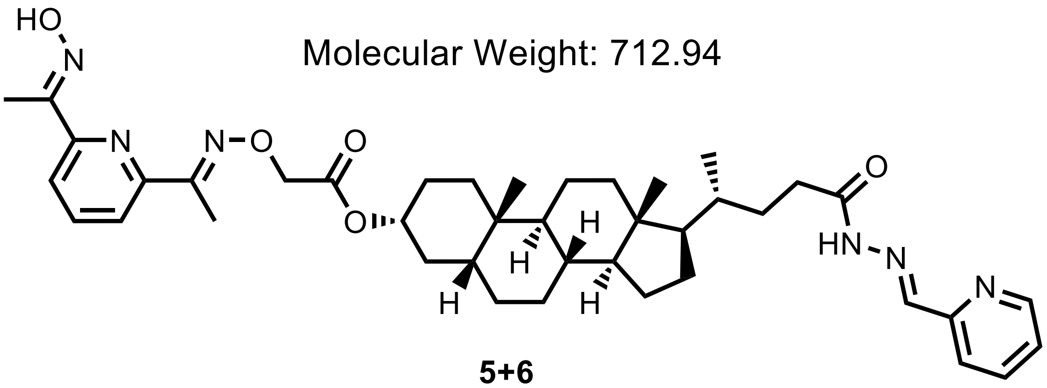

5+6

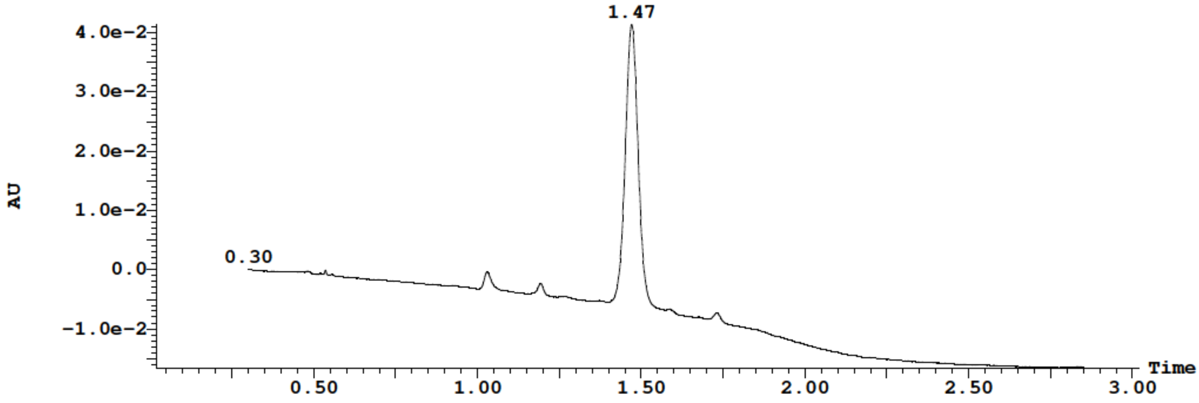

Peak ID	Compound	Time	Mass Found
8		1.49	Not Found

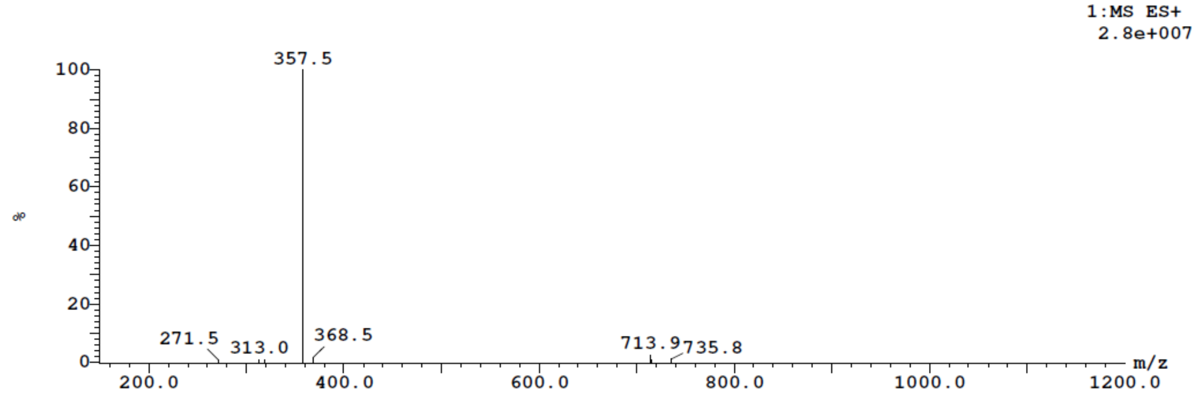

Figure S2. (top) HPLC-UV trace (280 nm) of formed hydrazone **6.** (bottom) MS chromatogram of peak identified at t=1.49 min.

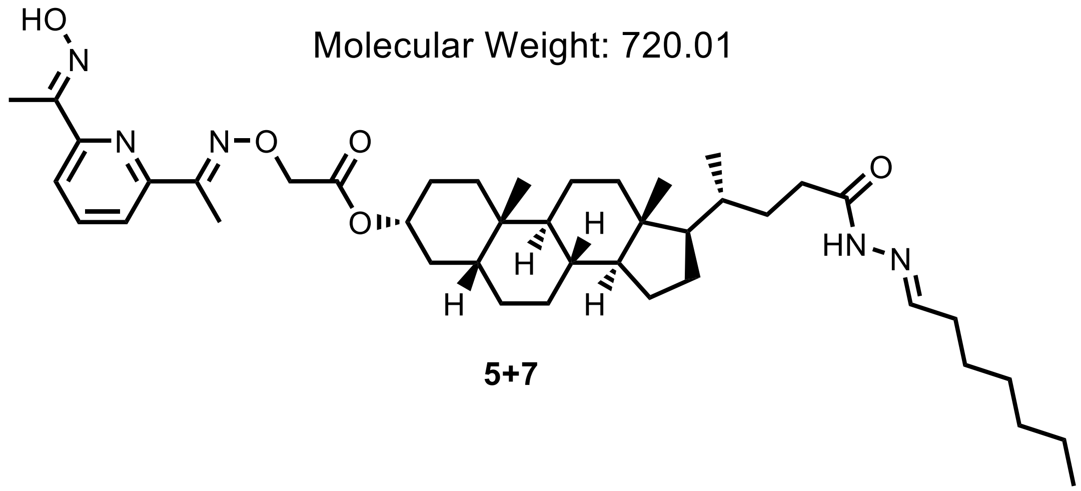

5+7

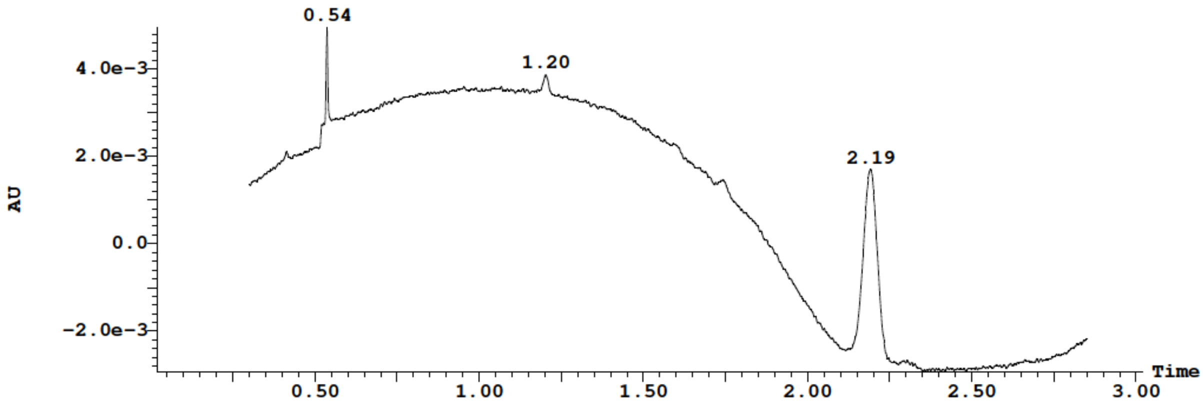

Figure S3. HPLC-UV trace (280 nm) of formed hydrazone **7**. No ionized species were detected.

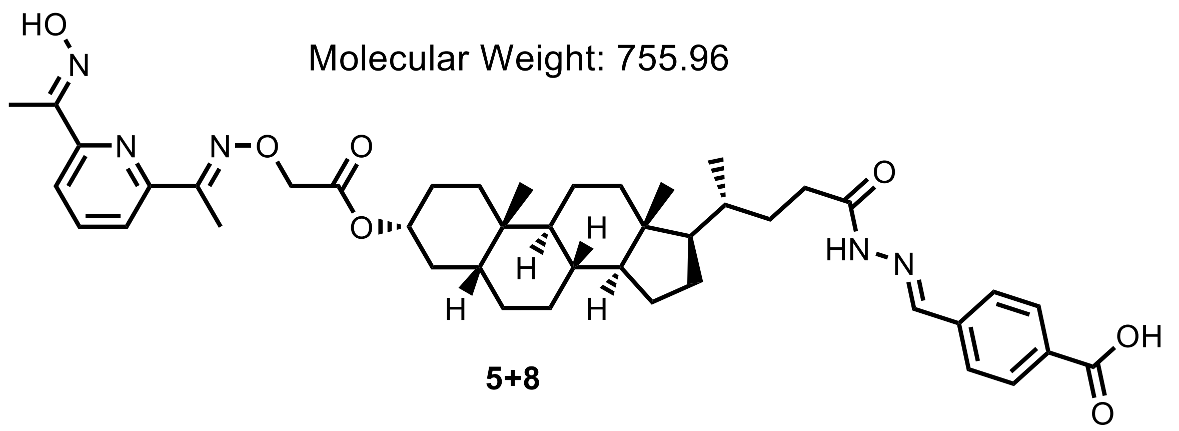

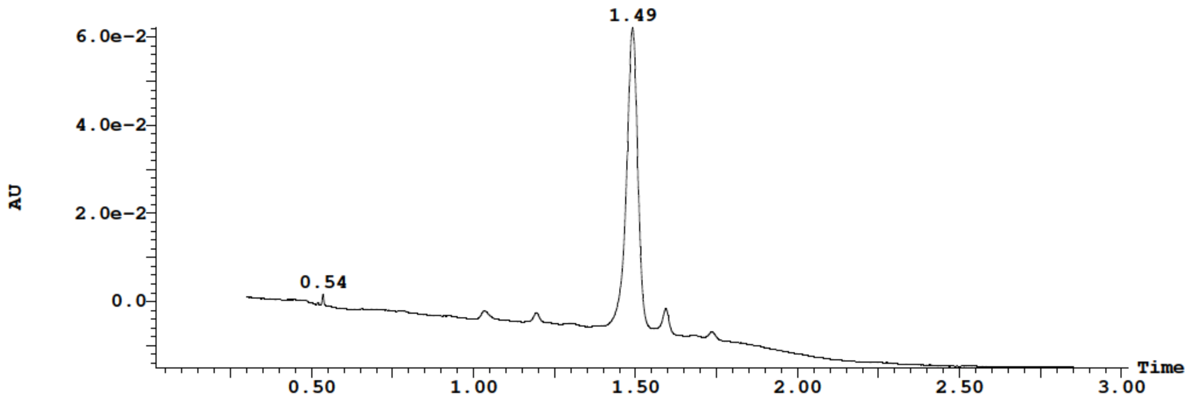

Peak ID	Compound	Time	Mass Found
15		1.51	Not Found

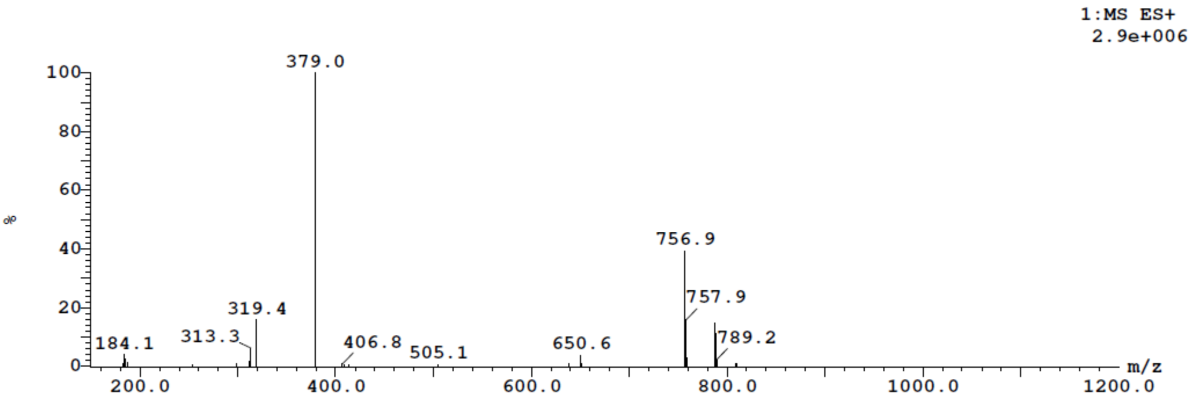

Figure S4. (top) HPLC-UV trace (280 nm) of formed hydrazone **8.** (bottom) MS chromatogram of peak identified at t=1.51 min

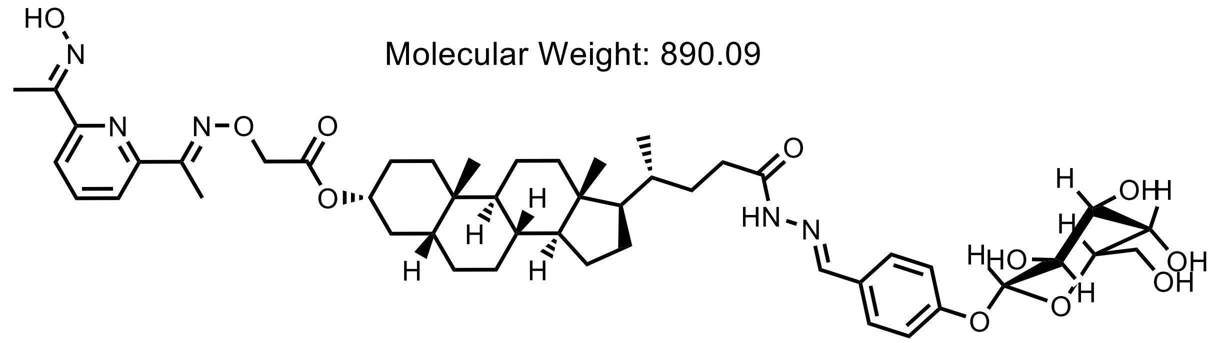

5+9

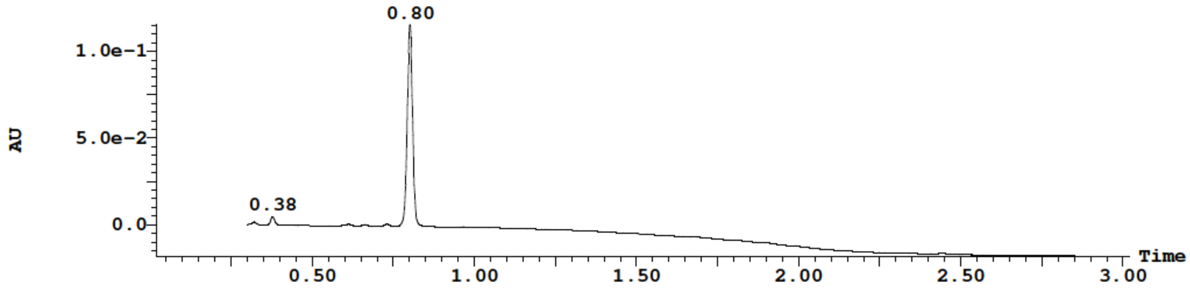

Peak ID	Compound	Time	Mass Found
7		0.82	Not Found

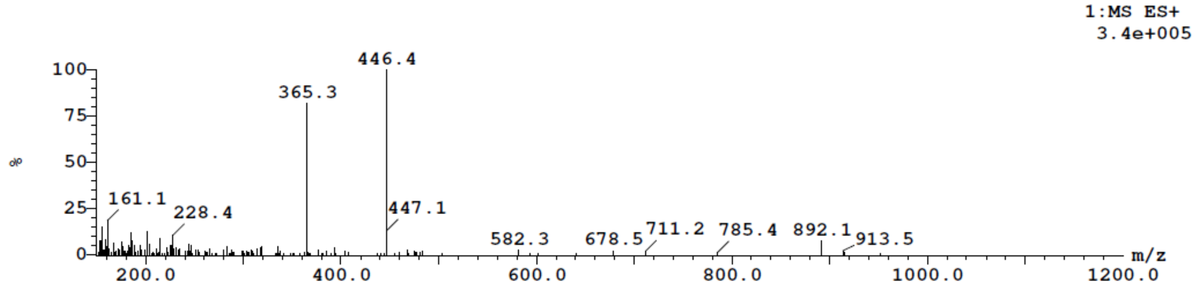

Figure S5. (top) HPLC-UV trace (280 nm) of formed hydrazone **9.** (bottom) MS chromatogram of peak identified at t=0.8 min

4. Ester Hydrolysis Experiments

Fluorescence spectroscopic data were recorded using a Cary Eclipse fluorescence spectrophotometer (Agilent). Fluorescence excitation experiments were recorded using the following parameters: emission wavelength = 510 nm, excitation range 380−480 nm, recorded at 4 minute intervals, with the excitation and emission slits set at 5 nm. All experiments were repeated a minimum of two times on independently prepared samples to confirm reproducibility. At the end of the experiment, 10 µL of 1M NaOH was added to hydrolyze all of the remaining Ac-HPTS substrate, and the emission measured at this end point was used to normalize the data.

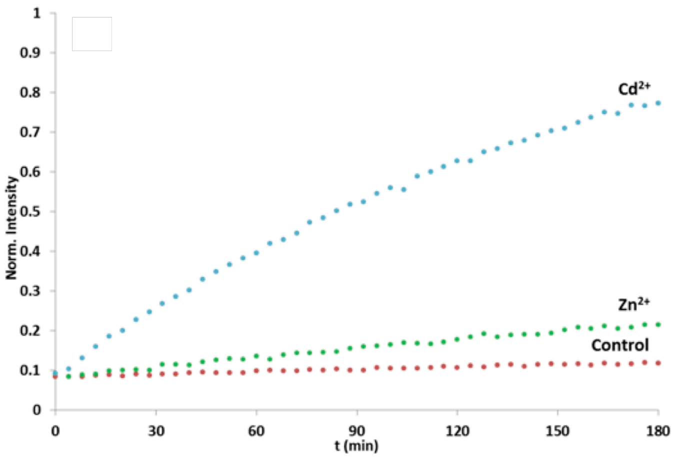

Figure S6. Time dependent normalized fluorescence intensity traces (Em=510 nm, Ex=415 nm) for Ac-HPTS hydrolysis. In bulk activity experiment: at t=0 min, 100 µM **5** was added to a solution containing 25 µM Ac-HPTS, 250 µM $CdSO_4$ or 250 µM $ZnCl_2$ in 50 mM MES buffer, pH=6.6, 100 mM NaCl, 0.05% Triton-x100. As a control, no $CdSO_4$ was used.

Dose response experiments:

Vesicles were prepared from 1,2-dioleoyl-sn-glycero-3-phosphocholine (DOPC chloroform solution). For vesicles containing the molecules, the corresponding amount was added to the lipid solution. The solvent was evaporated under a stream of nitrogen and the residue dried for 2h under high vacuum. The resulting thin film was hydrated in 500 µL of buffer solution (50 mM MES, 100 mM NaCl, pH=6.6) containing 250 µM Ac-HPTS. After hydration, the suspension was subjected to five freeze-thaw cycles (liquid nitrogen, water at room temperature). The obtained suspension was extruded 21 times through a 200 nm polycarbonate membrane. The vesicle suspension was separated from any extravesicular content by size exclusion chromatography using prepacked Sephadex G-25M columns (prequilibrated with buffer solution, as mobile phase). The obtained solution was diluted to the final concentration of 1 mM and used as stock solution within the day.

500 µL of stock vesicle solution (DOPC/**5** 97.5/2.5) was placed into a quartz fluorimetric cell. At t=30 min, $CdSO_4$ was injected from an aqueous stock solution at the corresponding concentration. At the end of the experiment, 10 µL of 5% Triton X-100 and 1M NaOH was added to lyse the vesicles and hydrolyze all of the remaining Ac-HPTS substrate. The emission measured at this end point was used to normalize the data taking into account of the dilution factor.

Hydrazone activity experiments:

500 µL of stock vesicle solution (DOPC/**5** 97.5/2.5) was placed into a quartz fluorimetric cell. At t=0 min, compounds were injected from a stock solution in DMSO. At t=30 min, 1 mM $CdSO_4$ was injected

from an aqueous stock. At the end of the experiment, 10 µL of 5% Triton X-100 and 1M NaOH was added to lyse the vesicles and hydrolyze all of the remaining Ac-HPT substrate. The emission measured at this end point was used to normalize the data taking into account of the dilution factor.

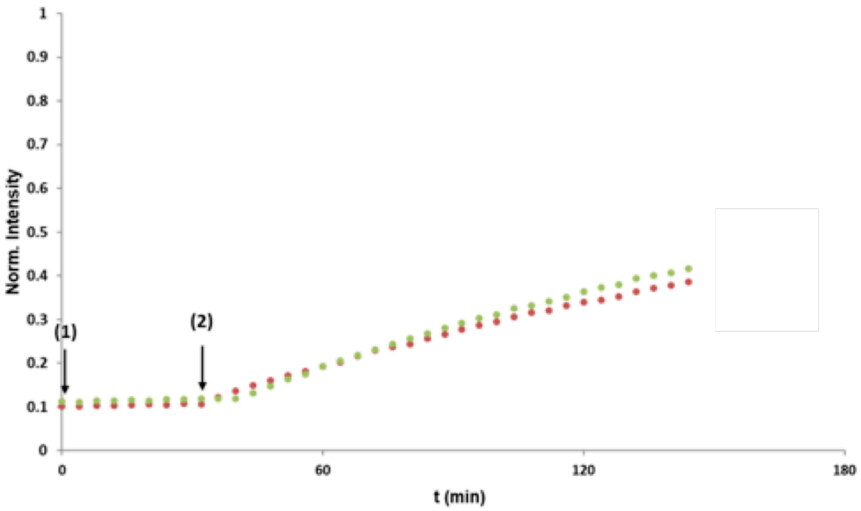

Figure S7. Effect of addition of **6** on the activity of membrane compound **4**. Red data: at t=30 min **(2)**, 1 mM $CdSO_4$ was added to a 1 mM vesicle solution containing 2.5 mol% **4**. Green data: at t=0 min **(1)**, 1 mM of **6** was added to a 1 mM vesicle solution containing 2.5 mol% **4**, and at t=30 min **(2)**, 1 mM $CdSO_4$ was added.

ON-OFF switching experiments:

500 µL of stock vesicle solution (DOPC/**5** 97.5/2.5) was placed into a quartz fluorimetric cell. At t=30 min, 1 mM **6** was injected from a stock solution in DMSO. At t=60 min, 1 mM $CdSO_4$ was injected. At t=75 min, 2 mM EDTA was injected. At t=130 min, 2 mM $CdSO_4$ was injected. At the end of the experiment, 10 µL of 5% Triton X-100 and 1M NaOH was added to lyse the vesicles and hydrolyze all of the remaining Ac-HPTS substrate. The emission measured at this end point was used to normalize the data taking into account of the dilution factor.

5. Cadmium Transport Experiments

Vesicles were prepared from 1,2-dioleoyl-sn-glycero-3-phosphocholine (DOPC chloroform solution). The solvent was evaporated under a stream of nitrogen and the residue dried for 2h under high vacuum. The resulting thin film was hydrated in 500 µL of buffer solution (10 mM MES, 100 mM $CdSO_4$, pH=6.6) containing 1 mM HPTS. After hydration, the suspension was subjected to five freeze-thaw cycles (liquid nitrogen, water at room temperature). The obtained suspension was extruded 21 times through a 200 nm polycarbonate membrane. The vesicle suspension was separated from any extravesicular content by size exclusion chromatography using prepacked Sephadex G-25M columns (prequilibrated with buffer solution, as mobile phase). The obtained solution was diluted to the final concentration of 0.1 mM and used as stock solution within the day.

500 µL of stock vesicle solution (DOPC) was placed into a quartz fluorimetric cell. Prior to the experiment, 5mol% of compound **5** or compound **5** and 1 mM 2-formylpyridine were injected and left to equilibrate for 30 mins. (formation of the conjugate was verified by LCMS-UV). At t = 1 min, a corresponding volume of aqueous H_2SO_4 (0.1 M) was injected in order to decrease the pH of the bulk solution to 6.1 (volume of needed H_2SO_4 solution was predetermined for each batch of vesicles using a SevenCompact pH/Ion S220 pH meter) The emission of HPTS at 510 nm was monitored at two excitation wavelengths (405 and 460 nm) simultaneously. Change in fluorescence was registered for 5 mins after which the vesicles are lysed with 10 µL 5% aqueous Triton X-100 solution to equilibrate the intra and extravesicular solution.

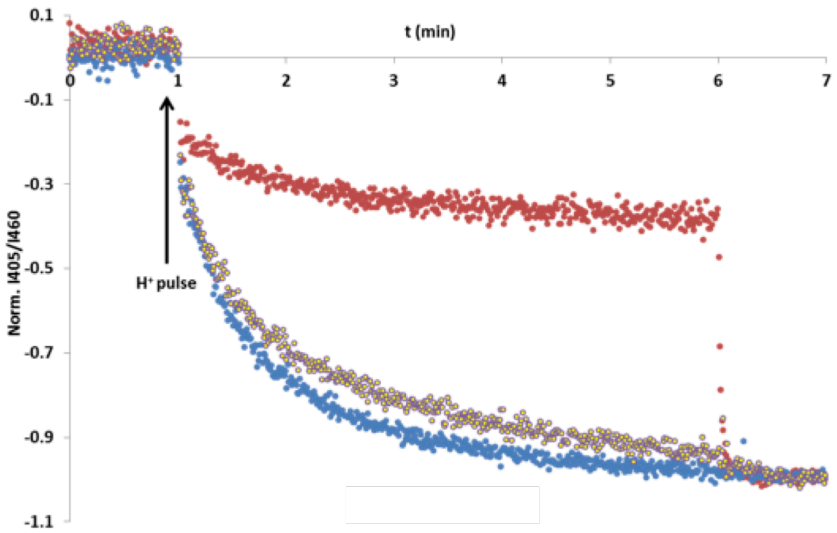

Figure S8. Comparison of the effect **5** (blue), the hydrazone conjugate formed by **5** and **6** (yellow) and of DMSO (red) on cadmium transport across vesicle bilayer membranes. DOPC vesicles containing 1 mM HPTS were prepared at pH 6.6 and suspended in 10 mM MES buffer with 100 mM $CdSO_4$ and DMSO (5 µl), **5** (5 mol% relative to lipids) or the hydrazone conjugate formed by **5** and **6** (5 mol% relative to lipids) were added. At t = 1 min., H_2SO_4 was added to lower the external pH to 6.1, and the local pH inside the vesicles was monitored using the ratio of the fluorescence emission at 510 nm due to the phenol (λ_{ex} = 405 nm) and phenolate (λ_{ex} = 460 nm) forms of HPTS. At t = 6 min., the vesicles were lysed by addition of Triton-x100 to obtain the HPTS emission at a pH of 6.1.

(1) Wang, P. S. P.; Nguyen, J. B.; Schepartz, A. Design and High-Resolution Structure of a B3-Peptide Bundle Catalyst. J. Am. Chem. Soc. 2014, 136 (19), 6810–6813. https://doi.org/10.1021/ja5013849.
